# Genomic characterization of non-CC398 methicillin-resistant *Staphylococcus aureus* from Belgian livestock reveals high similarity to human-associated lineages

**DOI:** 10.3389/fpubh.2026.1894809

**Published:** 2026-08-13

**Authors:** Bert Bogaerts, Carole Kowalewicz, Sigrid C. J. De Keersmaecker, Nancy H. C. Roosens, Kevin Vanneste, Cécile Boland

**Affiliations:** 1 Transversal Activities in Applied Genomics, Sciensano, Brussels, Belgium; 2 Veterinary Bacteriology, Sciensano, Brussels, Belgium

**Keywords:** MRSA, Staphylococcus aureus, livestock, whole-genome sequencing, One Health, HA-MRSA, antimicrobial resistance, non-CC398

## Abstract

**Introduction:**

Methicillin-resistant *Staphylococcus aureus* (MRSA) is a bacterial pathogen resistant to several widely used antibiotics. Although MRSA has historically been associated with hospital- and community-associated infections, livestock-associated MRSA (LA-MRSA) has emerged as a public health concern worldwide. While the majority of studies on LA-MRSA have focused on the CC398 lineage, little is known about the other lineages circulating in livestock. This study reports the whole-genome sequencing (WGS)-based characterization of 17 non-CC398 MRSA isolates with *spa* types typically associated with human infections.

**Methods:**

The isolates were collected from healthy food-producing farm animals in Belgium between 2011 and 2021. WGS was used for typing and detecting antimicrobial resistance (AMR), virulence genes, and plasmids. Core genome multi-locus sequence typing (MLST) and single nucleotide polymorphism (SNP)-based phylogenomic analyses were performed to investigate the relationship between isolates and to compare them with publicly available genomic data.

**Results:**

Five sequence types (STs) were identified: ST1 (*n* = 1), ST22 (*n* = 6), ST80 (*n* = 1), ST239 (*n* = 8), and ST612 (*n* = 1), all belonging to clonal complexes other than the typical livestock-associated CC398. For ST22 and ST239, we identified several isolates that showed very high genomic similarity, indicating either transmission between farms or acquisition from a common source. For ST239, we identified a shared lineage among isolates collected from laying hens and cattle throughout the study period. A comparison with public datasets revealed a high genomic similarity between the isolates from this study and globally circulating human-associated lineages, suggesting shared ancestry and clonal spread rather than direct evidence of zoonotic transmission.

**Discussion:**

This study provides insights into the genomic diversity, spread, and potential public health relevance of non-CC398 MRSA lineages circulating in Belgian livestock.

## 1 Introduction

Methicillin-resistant *Staphylococcus aureus* (MRSA) is a Gram-positive bacterium that frequently causes human diseases (1, 2). In humans, MRSA can infect the skin, soft tissues, bloodstream (bacteremia), lungs (pneumonia), and other parts of the body. MRSA infections have historically been divided into hospital-associated methicillin-resistant *Staphylococcus aureus* (HA-MRSA) and community-associated methicillin-resistant *Staphylococcus aureus* (CA-MRSA) infections, with substantial genomic and epidemiological differences (3). Furthermore, animals may play a role in transmitting and spreading MRSA (4). Livestock-associated MRSA (LA-MRSA) is an emerging public health problem in many parts of the world (2, 5). Transmission from humans to animals and vice versa have been reported (6–8). LA-MRSA is primarily associated with the CC398 lineage, which has been identified in pigs, cattle, poultry, and other animals, as well as in humans (4, 9, 10). Nevertheless, other MRSA lineages have also been reported to circulate in livestock animals, including CC5, CC9, and CC97, although these have been studied less extensively (11). In Belgium, non-CC398 MRSA isolates have been sporadically detected in food-producing animals since surveillance began in 2011 (12).

MRSA strains can express numerous virulence factors, including toxins, immunomodulators, and secreted enzymes (3, 13, 14), contributing to their adaptability and survival (3, 15). These factors span several functional categories. For example, proteases such as aureolysin (Aur) and serine protease-like proteins (SplA, SplB, and SplE) can enable tissue invasion and protein degradation. Immune evasion factors, including Sak, Scn, and EdinB, interfere with host defense mechanisms. MRSA can also express various cytotoxins and leukocidins (e.g., *γ*-hemolysin components and LukED/PVL) that contribute to host cell lysis, as well as a broad array of superantigens, including enterotoxins and the toxic shock syndrome toxin (encoded by the *tst* gene), which can drive immune activation and disease severity, including food poisoning in humans. Moreover, MRSA typically carries multiple mobile genetic elements (MGEs) such as plasmids (3, 13). The accessory component accounts for ~25% of the total genome size, contributing to the considerable genomic diversity and flexibility observed in MRSA (3). The pathotype of MRSA strains is highly correlated with their virulence gene content, and virulence gene characterization is, consequently, an important aspect of MRSA monitoring (16).

The emergence and spread of antimicrobial resistance (AMR) in *S. aureus* is a serious problem in public health and clinical settings (3, 17, 18). MRSA strains typically carry the staphylococcal chromosomal cassette (SCC*mec*), which contains either the *mecA* or *mecC* gene and confers resistance to most available β–lactam antibiotics (3). Although both genes mediate *β*-lactam resistance, *mecA* and *mecC* differ genetically and epidemiologically, sharing only ~70% sequence identity. The *mecC* gene is more commonly associated with non-human hosts and represents a potential zoonotic risk (19, 20). Currently, 15 types of the SCC*mec* cassette have been described, and characterization of this genomic region is commonly used to distinguish MRSA lineages (21, 22). MRSA strains frequently acquire resistance to other antibiotics as well through horizontal gene transfer and chromosomal point mutations. For example, strains that carry *dfrA* and *dfrK* (trimethoprim resistance), *erm(C)* (erythromycin resistance), *tet(L)*, or *tet(K)* (tetracycline resistance) on MGEs have been frequently reported (3, 18). Resistance to fluoroquinolones tends to be more stable since it is mainly associated with mutations in housekeeping genes such as *gyrA* and *parC* (also known as *grlA*) (23, 24). The high prevalence of AMR complicates the treatment of MRSA infections and requires continuous monitoring in light of the global AMR crisis (3, 25).

Multiple conventional techniques are used to characterize MRSA, including methods specific to *S. aureus* such as *spa* typing and SCC*mec* typing, in addition to universal methods such as multi-locus sequence typing (MLST) and PFGE (3, 21). These methods vary in cost and discriminatory power (3). Whole-genome sequencing (WGS) has become the preferred typing method, as it enables complete isolate characterization, including the detection of virulence genes, AMR genes and mutations, and other relevant genomic information. WGS also provides resolution to distinguish closely related strains. The added value of WGS for both outbreak scenarios and routine monitoring applications has extensively been demonstrated (14, 26, 27). WGS-based genomic databases such as RefSeq or AllTheBacteria facilitate the comparison with numerous historical cases (28). The AllTheBacteria database currently contains 121,427 *S. aureus* isolates, wherein the most frequent sequence types (STs) are ST8, ST5, and ST22. It also includes several thousand isolates belonging to CC398.

In this study, we used WGS to characterize MRSA isolates collected from healthy food-producing farm animals in Belgium between 2011 and 2021. The isolates belonged to *spa* types typically associated with human clinical cases rather than the classical LA-MRSA CC398 lineage. WGS was employed to analyze the presence of virulence genes, AMR genes, mutations, and plasmids. In addition, a phylogenomic analysis was performed to explore the relationship between the isolates and to compare them with publicly available genomes, including those collected from humans. This study aimed to characterize less well-studied non-CC398 MRSA lineages found in livestock animals and to investigate potential interspecies transmission, particularly between animals and humans.

## 2 Materials and methods

### 2.1 Isolate collection

MRSA isolates were selected from the voluntary Belgian AMR monitoring programs conducted between 2011 and 2021 and organized by the Federal Agency for the Safety of the Food Chain (29). All successfully recultured isolates available at the National Reference Laboratory for Antimicrobial Resistance (Sciensano, Belgium) that met the following selection criteria were included in this study: (1) *spa* types associated with either HA- or CA-MRSA lineages (11) and (2) not belonging to the livestock-associated clonal complex 398 (CC398), according to the PCR protocol described by Stegger et al. (30). During the study period, 3,928 pooled nasal swab samples were collected, yielding 1,053 MRSA isolates. This collection comprises all isolates among these that fulfilled the selection criteria. Non-CC398 MRSA isolates with HA-/CA-MRSA *spa* types corresponded to at most 4.3% of samples in any given year and animal category (dairy cattle, 2018) and accounted for 0.9% of all monitored samples overall (35/3,928). A detailed breakdown of the number of samples and isolates collected through the program, grouped by animal type, is provided in Supplementary Table S1. The isolates were assigned to *spa* types t037 (*n* = 8), t223 (*n* = 6), t044 (*n* = 1), t386 (*n* = 1), and t1257 (*n* = 1). The strains were isolated from nasal swabs collected from healthy food-producing farm animals as part of MRSA AMR monitoring. The swabs were collected from dairy cattle (*n* = 8), beef cattle (*n* = 5), laying hens (*n* = 2), calves (*n* = 1), and fattening pigs (*n* = 1). Farm location was recorded for all strains except two. All strains for which geographical information was available were collected from different farms. Since the geographical coordinates of these farms cannot be shared for privacy reasons, we grouped the isolates into geographical clusters by applying hierarchical ward clustering to the coordinates using the ‘AgglomerativeClustering’ function from scikit-learn v1.7.2 (Supplementary Table S2). The number of clusters (k = 5) was selected based on the average silhouette width and the total within-cluster sum of squares, which are shown in Supplementary Figure S1.

### 2.2 Antimicrobial susceptibility testing

Antimicrobial minimum inhibitory concentrations (MICs) were determined by broth microdilution using EUST (for the period 2011–2020) or EUST2 (for the year 2021) multiwell plates (Sensititre™; Thermo Fisher Scientific) and were interpreted based on epidemiological cut-off values (ECOFFs) according to the EFSA guidelines, as detailed in the annual reports of the Belgian National Reference Laboratory for antimicrobial resistance^1^.

### 2.3 Whole-genome sequencing

DNA samples were prepared from the bacteria grown on Columbia Sheep Blood Agar plates (Bio-Rad, Lokeren, Belgium) using the QIAGEN DNeasy Blood and Tissue Kit (QIAGEN, Hilden, Germany) following the manufacturer’s instructions for Gram-positive bacteria. Both DNA purity and concentration were assessed using NanoDrop 1000 (Isogen LifeScience, Utrecht, Netherlands). Sequencing libraries were created for each isolate using the Nextera XT DNA Library Preparation Kit (Illumina, San Diego, CA, USA) following the manufacturer’s instructions, and they were subsequently sequenced using MiSeq V3 chemistry (Illumina) for the production of 2 × 250 bp paired-end reads.

### 2.4 Data analysis

#### 2.4.1 Quality control and preprocessing

Raw reads were trimmed using fastp (31) v0.23.4 with the following options: ‘--detect_adapter_for_pe,’ ‘--cut_front,’ ‘--cut_front_window_size’ set to 1, ‘--cut_front_mean_quality’ set to 10, ‘--cut_tail,’ ‘--cut_tail_window_size’ set to 1, ‘--cut_tail_mean_quality’ set to 10, ‘--cut_right,’ ‘--cut_right_window_size’ set to 4, ‘--cut_right_mean_quality’ set to 20, and ‘--length_required’ set to 40. Processed reads were then *de novo* assembled using SPAdes (32) (v3.15.5), with the ‘--careful’ option enabled and ‘--cov-cutoff’ set to 10. Contigs smaller than 1,000 bp were removed using the seq function of seqtk v1.4 (available at https://github.com/lh3/seqtk). The quality of the assemblies was evaluated using QUAST (33) (v5.2.0), providing the filtered assembly as input. The mapping rate to the assembly was evaluated by mapping the processed reads against the filtered assembly using Bowtie2 (34) (v2.5.1), with the ‘--end-to-end’ and ‘--sensitive’ options enabled. Afterward, the ‘depth‘function of SAMtools (35) v1.17 was used with the ‘-a’ option enabled to calculate the median depth of the reads mapped to the assembly. Kraken 2 (36) v2.1.1 was used to screen for contamination using an in-house constructed database (See Supplementary Table S3). Datasets with >5% of reads assigned to species other than *S. aureus* were considered contaminated. Finally, datasets were screened for intra-species contamination using ConFindr (37) (v0.8.1), with the ‘--rmlst’ option enabled.

#### 2.4.2 Isolate characterization

The online SCCmecFinder (21) 1.2 tool was used to check for the presence of the *mecA* and *mecC* genes and to determine SCC*mec* types. Blastn (38) v2.14.0 was used with default options to determine *spa* types by aligning the contigs against the *spa* repeat sequences collected from the Ridom *spa* server (available at https://spaserver.ridom.de/). Sequence typing was performed using MiST (39) v1.0.0, with the *S. aureus* MLST and core genome MLST schemes retrieved from PubMLST.org (40) (accessed on 1st February 2026). The assembled contigs were screened for AMR genes using NCBI Antimicrobial Resistance Gene Finder (AMRFinderPlus) (41) (v4.0.19), with the ‘--coverage_min’ option set to 0.9 and database version ‘2025-07-16.1’. An additional AMR screening analysis was performed using ResFinder (42) (v4.4.2) on the assembled contigs, with the database downloaded on 25 May 2025 and the species option set to “*Staphylococcus aureus*.” The presence of genes encoding virulence factors was evaluated using the blastn-based gene detection workflow described previously (43), with the sequences from the VirulenceFinder (44) *Staphylococcus aureus* databases (accessed on 8 August, 2025). Only hits with >90% target coverage and >90% sequence identity were retained. The same methodology was used to screen contigs for plasmid replicons using the PlasmidFinder (45) database (accessed on 31 August 2025), with the minimum percent identity threshold increased to 95%. The ‘mob_recon’ command of MOB-suite (46) (v3.1.8) was used to predict if contigs originated from the chromosome or plasmids. These steps, starting from raw FASTQ data, are available as a stand-alone tool, the “Staphylococcus pipeline,” on our institute’s public Galaxy instance (registration required, https://galaxy.sciensano.be/) (47).

#### 2.4.3 Phylogenomic investigation

A core genome MLST allele matrix was constructed by combining the typing outputs and including only loci with perfect matches (i.e., 100% coverage and 100% identity). Subsequently, the datasets with <90% of alleles identified were removed from the allele matrix. Afterward, loci detected in <90% of datasets were removed. GrapeTree (48) v2.2 was then used to construct a minimum spanning tree from the filtered allele matrix, with the method parameter set to ‘MSTreeV2.’ Single nucleotide polymorphism (SNP)-based phylogenies were inferred for all detected sequence types (STs) with more than one isolate in this study. This analysis was performed using PACU (49) v1.0.0, which uses Gubbins (50) v3.3.1 for recombination filtering. All options were left at their default values. SNPs located in regions identified as (pro)phages by PHASTER (51) were excluded from the PACU SNP analysis by supplying the genomic coordinates to PACU via the ‘--bed-phages’ option. Minimum spanning trees were constructed from the SNP matrices generated by PACU using GrapeTree v2.2, with the method option set to ‘MSTreeV2.’

#### 2.4.4 Comparison with publicly available data

Sequence typing using the classic MLST scheme was performed for all *S. aureus* genomes (*n* = 121,427) in the AllTheBacteria database, release 2024–08 (28). Metadata for the corresponding BioSamples were retrieved from the associated SQLite database. Additional cgMLST-based minimum spanning trees were constructed for each ST observed in the in-house sequenced isolates. All corresponding isolates from AllTheBacteria were included, following the methodology described in section 2.4.3.

## 3 Results

### 3.1 WGS data quality

An overview of the generated WGS datasets and corresponding SRA accession numbers is provided in Supplementary Table S4. Read trimming statistics are provided in Supplementary Table S5. The median numbers of read pairs before and after trimming were 450,837 and 417,439, respectively. An overview of the assembly statistics is provided in Supplementary Table S6. The median numbers of contigs, N50, and total assembly length were 45, 154,970 bp, and 2,865,773 bp, respectively. The sequencing depth ranged from 27x to 82x, with a median of 66x. The mapping rate ranged from 98.88 to 99.51%, with a median of 99.12%. For all datasets, Kraken 2 did not classify more than 5% of reads as belonging to any species other than *S. aureus*, and ConFindr did not report intra-species contamination. These metrics indicate that the generated data were of high quality, and all datasets were retained for further analysis.

### 3.2 Isolate typing

The geographical clustering resulted in five groups, which are listed along with the mean distance to the cluster centers in Supplementary Table S2. Detected SCC*mec* types and accession numbers for the detected *mecA* genes are listed in Supplementary Table S7. All isolates carried the *mecA* gene, with three very similar variants occurring, corresponding to GenBank accession numbers AB037671 (*n* = 8), AB373032 (*n* = 8), and AB505628 (*n* = 1). All detected *mecA* sequences matched the reference sequences perfectly (i.e., 100% identity over the complete nucleotide sequence). The *mecC* gene was not detected in any of the datasets. Isolates were assigned to SCC*mec* type III (3A) (*n* = 8), IVa (2B) (*n* = 4), IV (2B) (*n* = 3), IVc(2B) (*n* = 1), and IVd (2B) (*n* = 1). The isolates in this study were assigned to five different sequence types: ST1 (*n* = 1), ST22 (*n* = 6), ST80 (*n* = 1), ST239 (*n* = 8), and ST612 (*n* = 1). The six ST22 isolates were all collected from cattle in 2018, including both dairy and meat animals. Five of the six isolates originated from the same geographical cluster. Geographical data were not available for the remaining isolate (VAR307). In contrast, the ST239 cluster contained isolates collected from cattle and hens between 2011 and 2021, originating from three of the five geographical groups.

### 3.3 Virulence gene detection

An overview of the detected virulence genes is provided in Table 1. Virulence gene profiles were identical for isolates assigned to the same ST. The number of detected virulence genes associated with the production of extracellular enzymes in each isolate varied between one and four. The *aur* gene was detected in all isolates. The *splA* and *splB* protease genes were detected in 11 of the 17 isolates, and they were absent in the 6 isolates assigned to ST22. The eight isolates assigned to ST239 additionally carried the *splE* gene. All isolates were found to contain the *sak* and *scn* genes, which are associated with the evasion of host innate immunity. The number of genes associated with the production of toxins varied between six and ten. The *hlgABC* operon was present in all isolates. The *lukD* and *lukE* genes were present in all isolates, except for the six isolates assigned to ST22. The *edinB* and *lukS-PV* genes were only observed in the isolate VAR698. All isolates carried one or more staphylococcal enterotoxin genes (i.e., *sea*, *seb*, *seg*, *seh*, *sei*, *sek*, *sem*, *sen*, *seo*, *seq*, and/or *seu*), except for the isolate VAR698. The ST22 isolates carried six *se* genes (*seg*, *sei*, *sem*, *sen*, *seo*, and *seu*), the ST239 isolates carried three genes (*sea*, *sek*, *seq*), the ST612 isolate carried four genes (*sea*, *seb*, *sek*, *seq*), and the ST1 isolate carried only *seh*. Lastly, the *tst* gene was present in the six isolates assigned to ST22.

**Table 1:** Detected virulence genes.

ST	Isolate	Exoenzymes				Host imm.		Toxins																		
		*aur*	*splA*	*splB*	*splE*	*sak*	*scn*	*edinB*	*hlgA*	*hlgB*	*hlgC*	*lukD*	*lukE*	*lukS-PV*	*sea*	*seb*	*seg*	*seh*	*sei*	*sek*	*sem*	*sen*	*seo*	*seq*	*seu*	*tst*
ST1	VAR708	1	1	1	0	1	1	0	1	1	1	1	1	0	0	0	0	1	0	0	0	0	0	0	0	0
ST22	VAR307	1	0	0	0	1	1	0	1	1	1	0	0	0	0	0	1	0	1	0	1	1	1	0	1	1
ST22	VAR699	1	0	0	0	1	1	0	1	1	1	0	0	0	0	0	1	0	1	0	1	1	1	0	1	1
ST22	VAR700	1	0	0	0	1	1	0	1	1	1	0	0	0	0	0	1	0	1	0	1	1	1	0	1	1
ST22	VAR701	1	0	0	0	1	1	0	1	1	1	0	0	0	0	0	1	0	1	0	1	1	1	0	1	1
ST22	VAR703	1	0	0	0	1	1	0	1	1	1	0	0	0	0	0	1	0	1	0	1	1	1	0	1	1
ST22	VAR704	1	0	0	0	1	1	0	1	1	1	0	0	0	0	0	1	0	1	0	1	1	1	0	1	1
ST80	VAR698	1	1	1	0	1	1	1	1	1	1	1	1	1	0	0	0	0	0	0	0	0	0	0	0	0
ST239	VAR304	1	1	1	1	1	1	0	1	1	1	1	1	0	1	0	0	0	0	1	0	0	0	1	0	0
ST239	VAR305	1	1	1	1	1	1	0	1	1	1	1	1	0	1	0	0	0	0	1	0	0	0	1	0	0
ST239	VAR697	1	1	1	1	1	1	0	1	1	1	1	1	0	1	0	0	0	0	1	0	0	0	1	0	0
ST239	VAR705	1	1	1	1	1	1	0	1	1	1	1	1	0	1	0	0	0	0	1	0	0	0	1	0	0
ST239	VAR706	1	1	1	1	1	1	0	1	1	1	1	1	0	1	0	0	0	0	1	0	0	0	1	0	0
ST239	VAR707	1	1	1	1	1	1	0	1	1	1	1	1	0	1	0	0	0	0	1	0	0	0	1	0	0
ST239	VAR785	1	1	1	1	1	1	0	1	1	1	1	1	0	1	0	0	0	0	1	0	0	0	1	0	0
ST239	VAR786	1	1	1	1	1	1	0	1	1	1	1	1	0	1	0	0	0	0	1	0	0	0	1	0	0
ST612	VAR702	1	1	1	0	1	1	0	1	1	1	1	1	0	1	1	0	0	0	1	0	0	0	1	0	0

Overview of the detected virulence genes in the in-house sequenced isolates. The first and second columns list the STs and isolate names, respectively. The subsequent columns indicate the detection of the corresponding gene. ‘1’ indicates that the gene was present, and ‘0’ indicates that the gene was absent. The colors correspond to the three categories of virulence genes defined in the VirulenceFinder database (44), as shown in the top row of the table. Immunity (‘imm.’).

### 3.4 AMR characterization

A complete overview of the genes and mutations associated with AMR detected by AMRFinder+ is provided in Supplementary Table S12. Genes associated with resistance to aminoglycosides were detected in all isolates except for the six isolates assigned to ST22. The eight isolates assigned to ST239 carried the *aadD1*, *ant(6)-Ia*, *ant(9)-Ia*, and *aph(3′)-IIIa* genes; isolates VAR698 (ST80) and VAR708 (ST1) carried the *ant(6)-Ia* and *aph(3′)-IIIa* genes, and the isolate VAR702 carried the *aac(6′)-Ie gene*. As mentioned in section 3.2, all isolates carried the *mecA* gene associated with resistance to β-lactam antibiotics. The eight ST239 isolates additionally carried the *mecR1* gene, which regulates the expression of *mecA*. The *mecI* gene, which downregulates the expression of *mecA*, was detected in all ST239 isolates, except VAR707. However, a premature stop codon was identified in all of these strains, which resulted in increased *mecA* expression (52). Several reads from the VAR707 isolate were also mapped to *mecI*, but coverage was insufficient to detect the gene and potential premature stop codon. Discrepancies in the presence of the *bla* genes between the isolates assigned to the same ST were investigated using a read mapping-based approach (Supplementary Figures S2–S5). For the ST1, ST22, and ST612 isolates, the detection of the *bla* genes was hindered by low local depth in the regions carrying these genes. The ST239 isolates likely contained two variants of these *bla* genes, as indicated by the higher depth and variant positions observed in the aligned reads. In addition, for some isolates, two alleles of the same AMR gene were detected on different contigs (unpublished results). Consequently, this resulted in issues with the *de novo* assembly, affecting the downstream AMR gene detection. The detection of these genes was updated based on visual inspection of the alignment of the processed reads against the gene sequences, confirming the presence of *blaI* (*n* = 16), *blaZ* (*n* = 16), *blaR1* (*n* = 10), and *blaPC1* (*n* = 16). VAR708 was the only isolate that carried the *pbp4* R200L mutation, which is also associated with resistance to β-lactam antibiotics. The *bleO* and *catA* genes, associated with resistance to bleomycin and phenicols, respectively, were detected in the eight isolates assigned to ST239. VAR708 (ST1) was the only isolate carrying the *fexA* gene, which is associated with resistance to phenicols. Genetic determinants associated with fosfomycin resistance were detected in the eight isolates assigned to ST239 and the one isolate assigned to ST612. These isolates contained the *fosB* gene and the *murA* G257D mutation. The *erm(A)* gene was detected in the eight isolates assigned to ST239. The *erm(C)* gene was detected in five isolates. Interestingly, the gene was only detected in three of the six ST22 isolates, and it was predicted to be plasmid-encoded. Similarly, the *qacC* gene, which is associated with resistance to quaternary ammonium compounds, was identified in two of the ST22 isolates (VAR701 and VAR703) and was predicted to be plasmid-encoded. VAR702 was the only isolate with the *gyrA* S84L and *parC* S80Y mutations associated with resistance to quinolones. In total, nine isolates, including the eight ST239 isolates, carried two mutations associated with rifamycin resistance in the *rpoB* gene. The *sat4* gene associated with resistance to streptothricin was detected in 10 isolates, corresponding to ST1, ST80, and ST239. Mutations in *folP* associated with sulfonamide resistance were detected in nine isolates, including E208K (*n* = 1), E258EKE (*n* = 8), and F17L (*n* = 9). All isolates carried the *tet(38)* gene. In addition, the tetracycline resistance genes *tet(K)* and *tet(M)* were both detected in nine isolates, including all eight ST239 isolates. Genetic determinants associated with trimethoprim resistance were detected in the isolate VAR702, which carried the *dfrS1* gene, as well as in the six ST22 isolates, which contained the *dfrB* A135T mutation. The ResFinder4 results are shown in Supplementary Table S8. These findings were largely consistent with the AMRFinder+ results, although fewer genes and mutations were detected by ResFinder4.

These genotypic AMR predictions were mostly consistent with the observed phenotypes (Supplementary Table S9). All isolates were resistant to cefoxitin, consistent with *mecA* carriage, and resistance to aminoglycosides, chloramphenicol, ciprofloxacin, erythromycin, rifamycins, sulfonamides, and tetracyclines co-occurred with the detected genes and/or mutations. Isolates that did not carry genetic resistance determinants for fusidic acid, linezolid, mupirocin, and vancomycin were susceptible to these agents. There were three notable exceptions: (1) although all isolates carried the intrinsic *tet(38)* efflux gene, tetracycline resistance was observed only in isolates that additionally carried acquired *tet(K)* and/or *tet(M)*, in line with the literature suggesting that the overexpression and not solely the presence of the *tet(38)* gene would contribute to the tetracycline resistant phenotype (53); (2) while *erm(A)* or *erm(C)* was associated with erythromycin resistance in all carriers, most appeared susceptible to clindamycin by MIC testing, consistent with an inducible MLSB phenotype that is not captured by MIC testing alone (54); and (3) the six ST22 isolates carrying the *dfrB* A135T substitution were phenotypically susceptible to trimethoprim, whereas the isolate carrying *dfrS1* was phenotypically resistant, indicating that *dfrB* A135T did not confer phenotypic trimethoprim resistance in this collection.

### 3.5 Plasmid characterization

The results of the MOB-suite plasmid screening are provided in Supplementary Table S10. In total, 21 putative plasmids were detected, varying from three to eight per isolate. Most detected plasmids were consistent across isolates with the same ST. However, there were numerous exceptions, for example, AD159 missing in VAR706 (ST239) or the presence of AA004 in VAR701 and VAR704 (ST22). The detected AMR genes that were located on contigs assigned to a putative plasmid are marked with ‘P’ in Supplementary Table S12. Of the 237 detected AMR genes, the majority (*n* = 136, 57.38%) were predicted to be plasmid-encoded. In contrast, none of the virulence genes was predicted to be plasmid-encoded. However, it should be noted that plasmid prediction from short-read data can be inaccurate due to fragmented assemblies and should be confirmed through other methods (55).

### 3.6 Phylogenomic investigation

**Figure 1:**
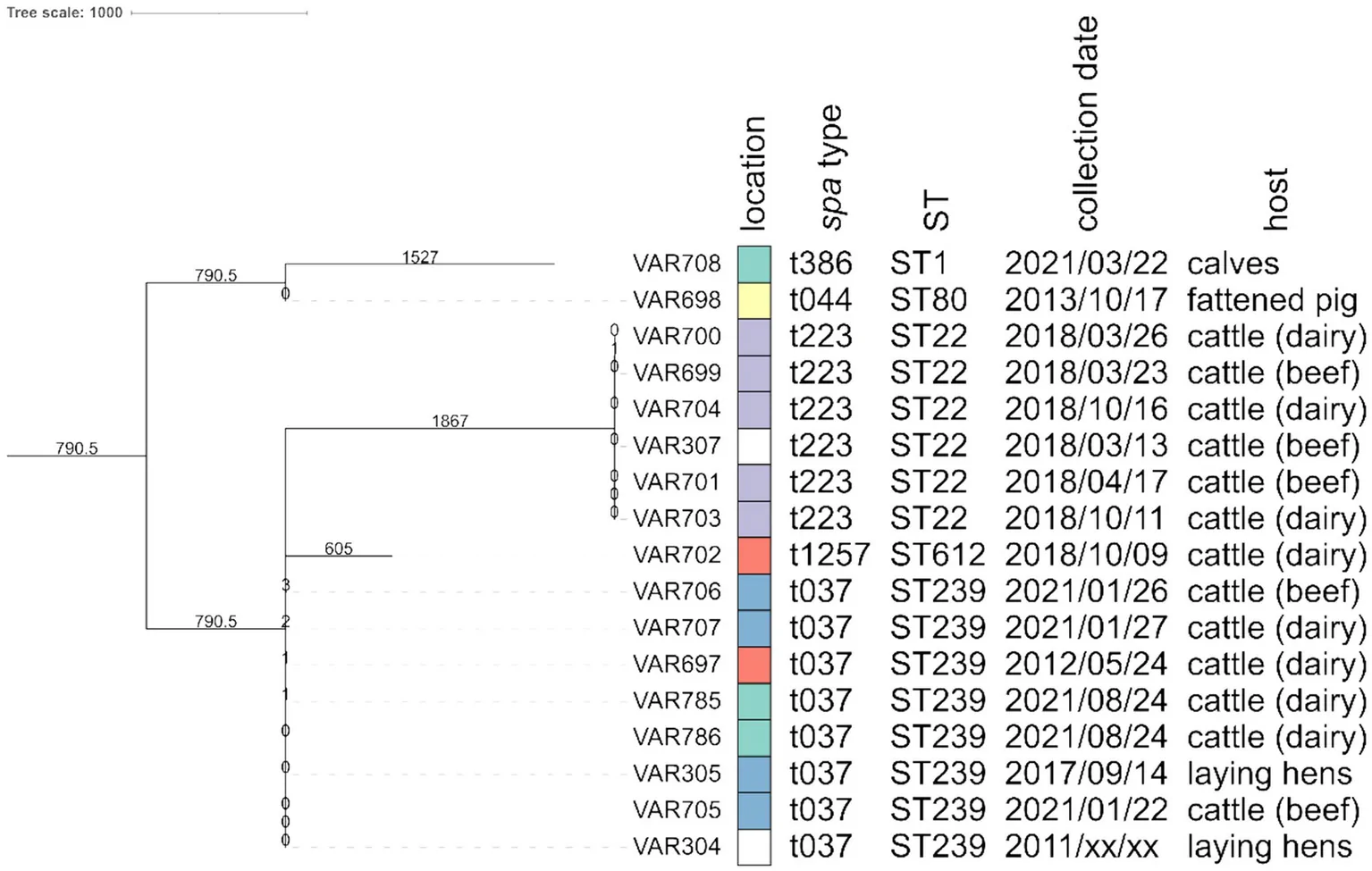
Core genome MLST phylogeny of the in-house sequenced isolates. The branch lengths correspond to the number of allele differences. The annotations are from left to right: Geographical cluster of the sampling location, *spa* type, sequence type (ST), collection date, and host. The colors of the location annotation correspond to Table S2, with the following mapping: Red (group A), turquoise (group B), purple (group C), blue (group D), and yellow (cluster E). The white box indicates that no geographical information was available. Isolate VAR304 was collected in 2011, but the exact date is unknown.

**Figure 2:**
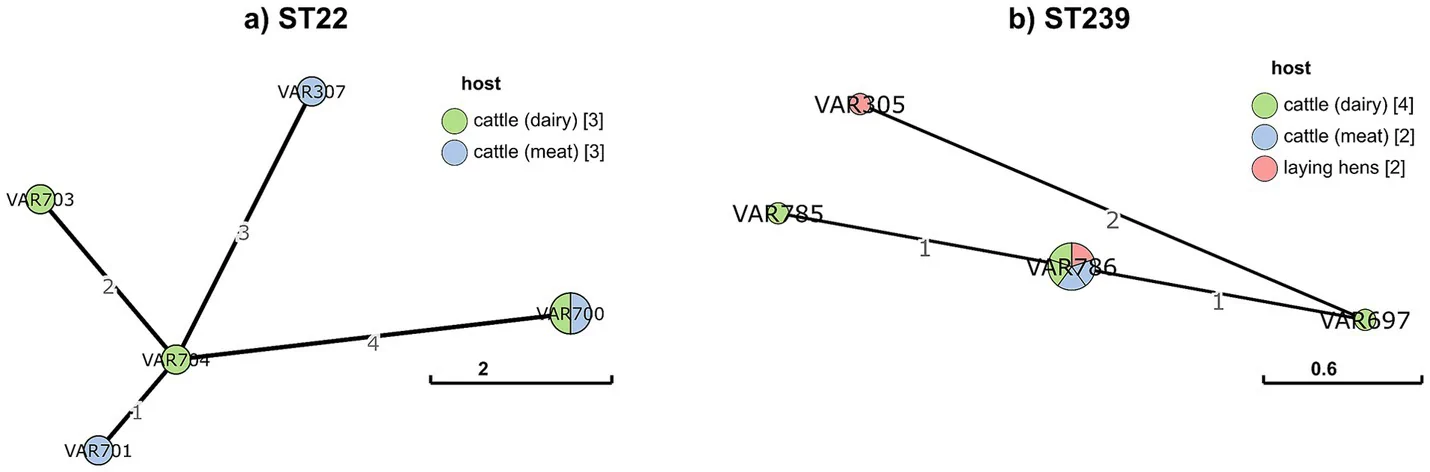
SNP-based phylogenies of the ST22 and ST239 in-house sequenced isolates. Minimum spanning trees were constructed from the core SNP matrices for ST22 **(a)** and ST239 **(b)**. Branch lengths and labels indicate the number of SNP differences between directly connected nodes. The sum of the distances across the branches does not necessarily equal the pairwise SNP distance between two isolates. Isolates are colored by host category (see legend). The following nodes were collapsed due to identical genotypes (no SNP differences): VAR699 and VAR700 in panel **(a)** and VAR304, VAR705, VAR706, VAR707, and VAR786 in panel **(b)**.

The allele matrix contained 2,075 of the 2,208 cgMLST loci after filtering. The resulting minimum spanning tree is shown in Figure 1. Isolates assigned to the same ST differed by 0–3 alleles, whereas large branch lengths existed between STs. Separate MSTs were constructed for the ST22 and ST239 isolates (Figure 2). The ST22 isolates differed by 0–4 SNPs and 0–1 alleles. This high level of genomic similarity, together with the fact that five of six isolates originated from the same geographical group despite originating from different farms (geographical data were not available for the sixth isolate) and that all six isolates were collected in 2018, suggests that they could be part of a single transmission chain. Notably, the isolates collected from beef (*n* = 3) and dairy (*n* = 3) cattle clustered closely together. The *erm(C)* gene was only present in the three isolates from the top clade (VAR701, VAR703, and VAR704) on high-depth contigs that were assigned to plasmid cluster AC627 by MOB-suite. Similarly, the *qac* gene was only present in isolates VAR701 and VAR703 on contigs assigned to plasmid cluster AB633 by MOB-suite. For both genes, the relevant contigs were identical in length and sequence across the carrier isolates and contained plasmid replication markers and, for AB633, relaxase markers, consistent with mobilizable plasmids. Furthermore, the substantially higher depth of the corresponding contigs is consistent with a high-copy-number plasmid, supporting the hypothesis that these genes can spread through plasmid transfer. However, short-read data are insufficient to reconstruct individual transfer events. No differences were observed for the other AMR genes detected in the isolates from this cluster. All isolates in the ST22 cluster had identical virulence gene profiles. For ST239, pairwise distances between the isolates ranged from 0 to 2 SNPs and 0–3 alleles. The resulting SNP-based phylogeny is shown in Figure 2B. Similar to ST22, this group of isolates included strains from meat and dairy cattle. However, it also contained two isolates collected from poultry. All isolates in this cluster had identical virulence gene and AMR profiles. Despite being collected from three geographical groups over a relatively long period of time (2011–2021), the isolates had a very high degree of genomic similarity. The location of one of these isolates was unknown.

### 3.7 Comparison with publicly available datasets

**Figure 3:**
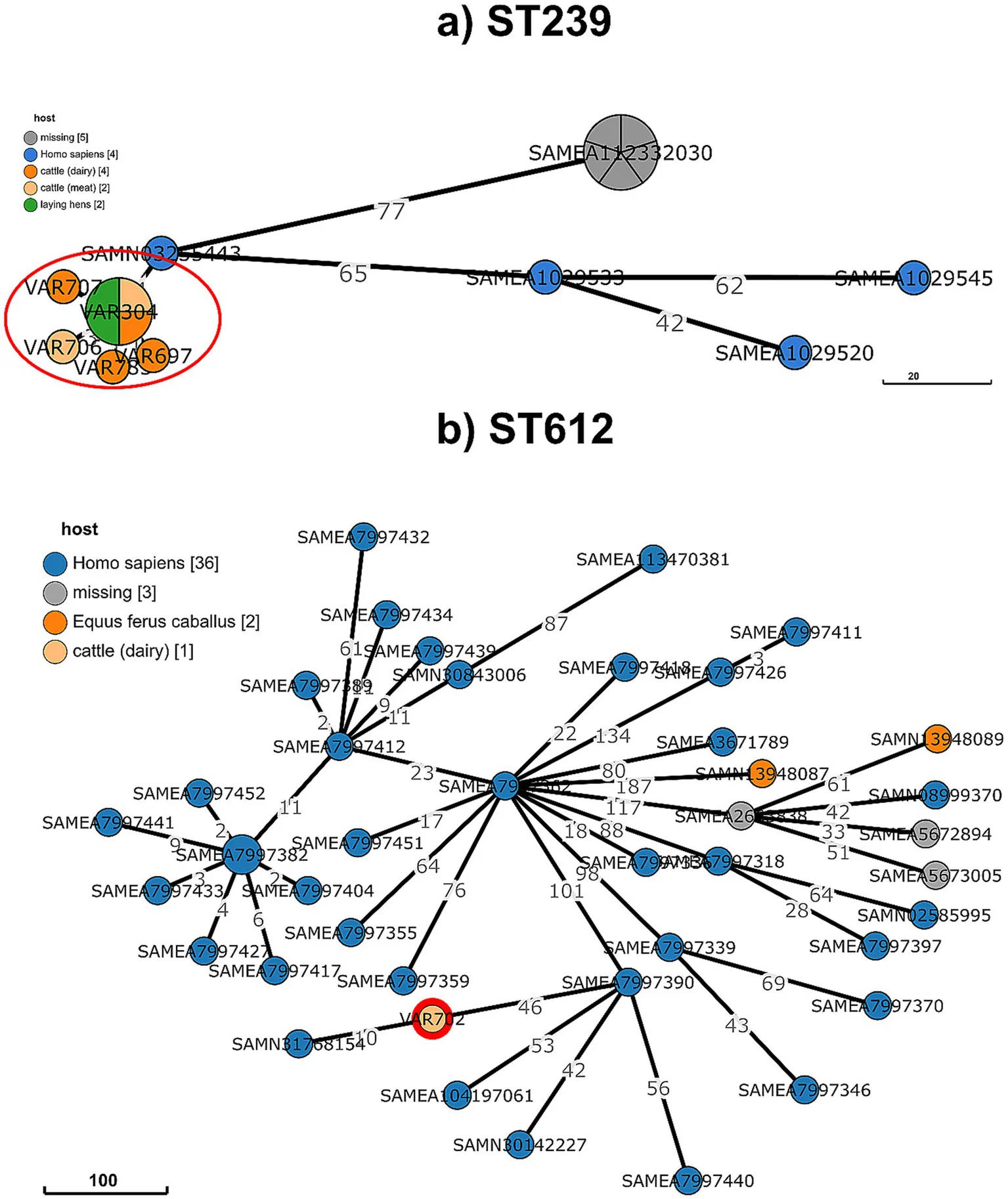
Core genome MLST phylogenies of ST239 and ST612 isolates using both in-house sequenced isolates and publicly available data. Core genome phylogeny of the isolates assigned to ST239 **(a)** and ST612 **(b)**. The colors correspond to host species (see legend). Branch lengths correspond to the number of allelic differences and were scaled logarithmically. The in-house isolates are highlighted in red. Note that the visualization of ST239 only includes nine of the 3,086 ST239 isolates in the AllTheBacteria database. These isolates were selected based on their relatively small allele distances from the in-house isolate.

The number of matching assemblies from the AllTheBacteria database for the five STs observed in this study that passed QC ranged from 41 for ST612 to 12,095 for ST22, as indicated in Supplementary Table S11. Across all observed STs, the majority of isolates originated from human hosts, although host information was not available for every strain. Separate core genome phylogenies were constructed for each ST. Close genomic relatedness between isolates was defined as clustering within 10 alleles, as used elsewhere (56). Evidence of close genomic relatedness between isolates from this study and publicly available datasets was observed for ST239 and ST612 (Figure 3). In contrast, for the other STs, the closest public isolate differed by more than 10 alleles (Supplementary Figures S6–S9). The phylogeny of the ST239 isolates from this study together with those from the public datasets is shown in Figure 3A. The in-house datasets exhibited high genomic similarity to SAMN03255443, with a minimum allele distance of four. This isolate was collected from human blood in the USA in 1981, but disease information is not available. All of the publicly available ST239 isolates were collected from humans (n = 1,832), except for one isolate that was obtained from an animal of an unspecified species. However, this isolate did not show a high degree of similarity to any of the isolates collected in this study. Host information was unavailable for a substantial proportion of the dataset (*n* = 1,253). Figure 3B shows the ST612 phylogeny. The VAR702 isolate, which was collected during this study, was located in a sub-clade with five other isolates, all of which were collected from humans. The most similar isolate was SAMN31768154, which was collected from a human in the Netherlands in 2021, with a distance of 10 alleles. However, disease information was not available for this isolate either. Interestingly, the VAR702 isolate had an identical virulence gene profile to the other five isolates in this clade. This profile was also the most prevalent among all ST612 isolates. For ST1 and ST22, the most closely related strains were quite distant, with 80 and 38 allele differences, respectively. Cows were the second most common host for ST1 isolates after humans, with 253 and 2,803 isolates, respectively. However, host information was often missing (*n* = 1,446). Interestingly, while several clades consisted mainly of isolates collected from cows, the VAR708 isolate (obtained from a pool of calves) clustered within a clade that was almost entirely composed of human isolates (Supplementary Figure S7). ST22 comprised a large number of isolates. The most common host by far was humans (*n* = 5,481), followed by dogs (*n* = 61), although host information was missing for many strains (*n* = 6,526). The in-house isolates were most closely related to human isolates, albeit at a relatively large distance, with the most similar isolate differing by 48 alleles. For VAR698, assigned to ST80, no closely related strains were identified in the public data. The most similar isolate differed by 45 alleles. Of the 41 ST612 isolates from AllTheBacteria, the vast majority were collected from humans (*n* = 36), while host information was missing for 3 isolates. The only other isolates obtained from a non-human host were two isolates collected from horses.

## 4 Discussion

This study describes the WGS-based characterization of 17 MRSA strains collected from healthy food-producing farm animals with *spa* types historically associated with human infection. All strains carried various genetic elements associated with AMR and virulence. The phylogenomic analysis revealed two groups of genomically highly similar isolates, indicating either transmission between farms or acquisition from a common source. For ST239, this group of closely related isolates included isolates from both laying hens and cattle, indicating the circulation of a shared lineage across host species. The comparison of our isolates with publicly available datasets revealed high genomic similarity between several of our isolates and human-associated lineages. This similarity is most consistent with shared ancestry and clonal circulation of these lineages rather than direct animal-to-human or human-to-animal transmission, which was not observed in this study.

The detected virulence genes were identical for isolates with the same ST. In total, 25 different virulence genes from the *Staphylococcus* VirulenceFinder database were identified across all isolates. The ST80 VAR698 isolate had an atypical virulence gene and AMR profile compared to other isolates. This was the only isolate that did not contain any staphylococcal enterotoxin genes associated with food poisoning (14). In addition, it was the sole isolate carrying *edinB* and *lukS-PV*. The *lukS-PV* gene encodes Panton–Valentine leukocidin (PVL), which is associated with skin and soft tissue infections, while *edinB* is associated with invasive infection (57, 58). Our findings are consistent with a previous large-scale review of MRSA ST80 strains, which reported that the majority of strains are PVL-positive and associated with *spa* type t044 (59). The *lukD* and *lukE* genes, encoding a different leukocidin, were detected in all isolates except for the ST22 isolates (60). The ST22 isolates were the only ones to carry the *tst* gene. This gene, which is associated with toxic shock syndrome, has been previously documented in ST22 strains identified in healthy children (61, 62). All strains carried several genes or mutations associated with AMR. In contrast to virulence genes, some differences were observed between isolates assigned to the same ST. For the ST22 cluster, there were indications that the *ermC* and *qacC* genes, associated with resistance to macrolide antibiotics and quaternary ammonium compounds, respectively, had been acquired through plasmid transfer. Many other AMR genes were also located on contigs predicted to originate from plasmids (Supplementary Table S12), suggesting that AMR genes can be relatively easily transferred between strains.

We identified a cluster of six very closely related isolates assigned to ST22, which is frequently observed in hospital settings (63, 64). However, studies have shown that this ST also occurs in bovine milk (65), as well as in wild swine, primates, dogs, and other animals (7, 66). At the time of writing, the AllTheBacteria database contained 12,095 strains assigned to ST22, with the large majority originating from humans. Only a handful of these isolates were collected from livestock. Five of the six ST22 isolates in our study were in the same geographical cluster (the location of the sixth isolate was unknown) and were collected within the same year, consistent with a potential point-source outbreak. However, as only one isolate was available per farm, it was not possible to distinguish between farm-to-farm transmission and independent acquisition from a shared common source. These isolates originated from dairy and beef cattle, suggesting that the same lineage is present in different cattle production systems, although they are not usually in direct contact.

A second cluster of eight closely related ST239 isolates was identified. ST239 corresponds to another relatively common HA-MRSA clone, which has spread globally over the last 50 years (67), and has been reported previously in healthy chickens in Belgium (68). The AllTheBacteria database contained 3,086 strains assigned to ST239, all but one originating from humans. However, host information was missing for 1,253 of these isolates. While ST239 has been identified previously in bovine milk and chickens, genomics datasets remain scarce, limiting our understanding of the spread of this clone in the veterinary sector (68, 69). The isolates in this cluster showed high genomic similarity to a publicly available *S. aureus* isolate obtained from a human infection. However, as this isolate was collected in the USA in 1981, direct transmission is highly unlikely. The observed similarity is, therefore, more consistent with shared ancestry within a human-associated lineage. It is also possible that more closely related isolates, including geographically and temporally intermediate strains or isolates from intermediate hosts, have not been sampled or are not currently represented in public genomic databases. In contrast to the ST22 cluster, the isolates were collected from different host types (both cattle and hens) and multiple geographical clusters over a longer period (2011 to 2021), suggesting that this clone circulates latently in a broad population and wider geographical area. The ST239 isolates carried genetic determinants associated with resistance to 10 classes of antibiotics, more than any other isolate in this study. No differences were observed in the AMR and virulence gene profiles of the isolates in this cluster.

The remaining three isolates were assigned to ST1, ST80, and ST612. ST1 first emerged as a CA-MRSA lineage but has since also been reported in hospital settings, companion animals, food products, and livestock (70–72). The wide variety of hosts suggests that this clone can easily adapt itself to different host species (72). While MRSA ST80 has been reported previously in veterinary animals (73), there is currently a lack of genomic data to understand the spread of this strain. The VAR698 isolate showed high genomic similarity to several genomes collected from human cases, including patients with disease (Supplementary Figure S9). Lastly, ST612 has been reported as the dominant clone in South African hospitals (74). The ST612 isolate collected in this study (i.e., VAR702) showed high genomic similarity to a human isolate collected in the Netherlands in 2021. The presence of MRSA strains from different STs that are genomically highly similar to human pathogenic clones suggests that these strains could be transmitted between humans and animals, and vice versa. Across all STs, genotypic AMR predictions were mostly consistent with phenotypic susceptibility testing for the majority of antimicrobial classes (Supplementary Table S9). The exceptions, including the intrinsic *tet(38)* efflux gene and the inducible clindamycin phenotype of the isolates carrying the *erm* gene, reflect well-described features of these determinants rather than true genotype–phenotype mismatches (54, 75).

There are several limitations of our study that need to be considered. First, we only had access to isolates collected from animal hosts, and any direct potential links to human infection could, therefore, not be identified. We were able to partially resolve this through the inclusion of public datasets collected primarily from human cases. However, we could not provide evidence for the direct transmission of MRSA between humans and farm animals. Sampling people in direct contact with livestock could have increased the likelihood of finding a link, but this was unfortunately not possible. Second, we only had access to a single isolate per farm, and we, consequently, could not assess the genetic diversity that potentially exists within MRSA strains circulating at the same farm. Third, the public databases are overrepresented with isolates collected from humans, and important contextual information for veterinary isolates may be lacking. Studies such as this one aim to address this gap, which is crucial to understand the spread of MRSA within a One Health context. Fourth, given the small number of isolates belonging to these lineages collected through historical monitoring, it was less likely that epidemiological links would be identified, both among the isolates in this study and when compared with publicly available data.

In conclusion, we report the characterization of MRSA strains from healthy food-producing farm animals, several of which showed high genomic similarity to strains collected from human infections. Due to their extensive repertoire of virulence genes and antimicrobial resistance (AMR) determinants, these isolates may pose a public health threat. For ST22 and ST239, we observed clusters of closely related isolates, indicating a potential transmission between farms and, in the case of ST239, even between hosts of different species. In addition, our results suggest that some clones may circulate latently over a relatively long period in a broad population and large geographical areas.

## Data Availability

The datasets presented in this study can be found in online repositories. The names of the repository/repositories and accession number(s) can be found https://www.ncbi.nlm.nih.gov/, PRJNA1443700.

## Footnotes

1
https://favv-afsca.be/fr/antibioresistance-resultats
