# Supplementary material for: Genomic characterization of non-CC398 methicillin-resistant Staphylococcus aureus from Belgian livestock reveals high similarity to human-associated lineages
Source: Front Public Health. 2026 Aug 13;14:1894809. doi: 10.3389/fpubh.2026.1894809 (PMC13518318; doi:10.3389/fpubh.2026.1894809)
Supplement: Supplementary file 1 [file Supplementary_file_1.docx]

SUPPLEMENTARY MATERIAL

**Genomic characterization of non-CC398 Methicillin-Resistant Staphylococcus**

**aureus from Belgian livestock reveals high genomic similarity to human-associated lineages**

# Tables

## Table S1: Samples and MRSA isolates from the Belgian AMR monitoring program (2011–2021)

| **Animal type** | **Category** | **2011** | **2012** | **2013** | **2014** | **2015** | **2016** | **2017** | **2018** | **2019** | **2020** | **2021** |
| --- | --- | --- | --- | --- | --- | --- | --- | --- | --- | --- | --- | --- |
| Veal calves | Samples | NT | 104 | NT | NT | 147 | NT | NT | 145 | NT | NT | 145 |
|  | MRSA isolates | NT | 49 | NT | NT | 116 | NT | NT | 79 | NT | NT | 79 |
|  | - CC398 MRSA isolates | NT | 48 | NT | NT | 87 | NT | NT | 79 | NT | NT | 78 |
|  | - non-CC398 MRSA isolates | NT | 0 | NT | NT | 28 | NT | NT | 0 | NT | NT | 1 |
|  | - non-CC398 isolates with LA-MRSA *spa*-types | NT | 0 | NT | NT | 22 | NT | NT | 0 | NT | NT | 0 |
|  | - non-CC398 isolates with HA/CA MRSA *spa* types, not recovered | NT | 0 | NT | NT | 6 | NT | NT | 0 | NT | NT | 0 |
|  | - non-CC398 isolates with HA/CA MRSA *spa* types, successfully re-cultured | NT | 0 | NT | NT | 0 | NT | NT | 0 | NT | NT | 1 |
|  | - Untypable MRSA isolates | NT | 1 | NT | NT | 1 | NT | NT | 0 | NT | NT | 0 |
| Dairy cows | Samples | NT | 141 | NT | NT | 96 | NT | NT | 93 | NT | NT | 128 |
|  | MRSA isolates | NT | 14 | NT | NT | 10 | NT | NT | 13 | NT | NT | 15 |
|  | - CC398 MRSA isolates | NT | 11 | NT | NT | 8 | NT | NT | 9 | NT | NT | 12 |
|  | - non-CC398 MRSA isolates | NT | 2 | NT | NT | 1 | NT | NT | 4 | NT | NT | 3 |
|  | - non-CC398 isolates with LA-MRSA *spa*-types | NT | 0 | NT | NT | 1 | NT | NT | 0 | NT | NT | 0 |
|  | - non-CC398 isolates with HA/CA MRSA *spa* types, not recovered | NT | 1 | NT | NT | 0 | NT | NT | 0 | NT | NT | 0 |
|  | - non-CC398 isolates with HA/CA MRSA *spa* types, successfully re-cultured | NT | 1 | NT | NT | 0 | NT | NT | 4 | NT | NT | 3 |
|  | - Untypable MRSA isolates | NT | 1 | NT | NT | 1 | NT | NT | 0 | NT | NT | 0 |
| Beef cattle | Samples | NT | 187 | NT | NT | 104 | NT | NT | 103 | NT | NT | 85 |
|  | MRSA isolates | NT | 19 | NT | NT | 16 | NT | NT | 9 | NT | NT | 4 |
|  | - CC398 MRSA isolates | NT | 18 | NT | NT | 13 | NT | NT | 6 | NT | NT | 2 |
|  | - non-CC398 MRSA isolates | NT | 1 | NT | NT | 3 | NT | NT | 3 | NT | NT | 2 |
|  | - non-CC398 isolates with LA-MRSA-*spa*-types | NT | 0 | NT | NT | 3 | NT | NT | 0 | NT | NT | 0 |
|  | - non-CC398 isolates with HA/CA MRSA *spa* types, not recovered | NT | 1 | NT | NT | 0 | NT | NT | 0 | NT | NT | 0 |
|  | - non-CC398 isolates with HA/CA MRSA *spa* types, successfully re-cultured | NT | 0 | NT | NT | 0 | NT | NT | 3 | NT | NT | 2 |
|  | - Untypable MRSA isolates | NT | 0 | NT | NT | 0 | NT | NT | 0 | NT | NT | 0 |
| Breeding pigs | Samples | NT | NT | NT | NT | NT | 153 | NT | NT | 179 | NT | NT |
|  | MRSA isolates | NT | NT | NT | NT | NT | 91 | NT | NT | 83 | NT | NT |
|  | - CC398 MRSA isolates | NT | NT | NT | NT | NT | 64 | NT | NT | 83 | NT | NT |
|  | - non-CC398 MRSA isolates | NT | NT | NT | NT | NT | 10 | NT | NT | 0 | NT | NT |
|  | - non-CC398 isolates with LA-MRSA-*spa*-types | NT | NT | NT | NT | NT | 10 | NT | NT | 0 | NT | NT |
|  | - non-CC398 isolates with HA/CA MRSA *spa* types, not recovered | NT | NT | NT | NT | NT | 0 | NT | NT | 0 | NT | NT |
|  | - non-CC398 isolates with HA/CA MRSA *spa* types, successfully re-cultured | NT | NT | NT | NT | NT | 0 | NT | NT | 0 | NT | NT |
|  | - MRSA isolates, not typed | NT | NT | NT | NT | NT | 17 | NT | NT | 0 | NT | NT |
| Fattening pigs | Samples | NT | NT | NT | NT | NT | 177 | NT | NT | 180 | NT | NT |
|  | MRSA isolates | NT | NT | NT | NT | NT | 112 | NT | NT | 105 | NT | NT |
|  | - CC398 MRSA isolates | NT | NT | NT | NT | NT | 77 | NT | NT | 87 | NT | NT |
|  | - non-CC398 MRSA isolates | NT | NT | NT | NT | NT | 24 | NT | NT | 0 | NT | NT |
|  | - non-CC398 isolates with LA-MRSA-*spa*-types | NT | NT | NT | NT | NT | 23 | NT | NT | 0 | NT | NT |
|  | - non-CC398 isolates with HA/CA MRSA *spa* types, not recovered | NT | NT | NT | NT | NT | 1 | NT | NT | 0 | NT | NT |
|  | - non-CC398 isolates with HA/CA MRSA *spa* types, successfully re-cultured | NT | NT | NT | NT | NT | 0 | NT | NT | 0 | NT | NT |
|  | - MRSA isolates, not typed | NT | NT | NT | NT | NT | 11 | NT | NT | 18 | NT | NT |
| Pigs unspecified | Samples | NT | NT | 328 | NT | NT | NT | NT | NT | NT | NT | NT |
|  | MRSA isolates | NT | NT | 215 | NT | NT | NT | NT | NT | NT | NT | NT |
|  | - CC398 MRSA isolates | NT | NT | 201 | NT | NT | NT | NT | NT | NT | NT | NT |
|  | - non-CC398 MRSA isolates | NT | NT | 3 | NT | NT | NT | NT | NT | NT | NT | NT |
|  | - non-CC398 isolates with LA-MRSA-*spa*-types | NT | NT | 0 | NT | NT | NT | NT | NT | NT | NT | NT |
|  | - non-CC398 isolates with HA/CA MRSA *spa* types, not recovered | NT | NT | 2 | NT | NT | NT | NT | NT | NT | NT | NT |
|  | - non-CC398 isolates with HA/CA MRSA *spa* types, successfully re-cultured | NT | NT | 1 | NT | NT | NT | NT | NT | NT | NT | NT |
|  | - MRSA isolates, not typed | NT | NT | 11 | NT | NT | NT | NT | NT | NT | NT | NT |
| Laying hens | Samples | 531 | NT | NT | 246 | NT | NT | 236 | NT | NT | 28 | NT |
|  | MRSA isolates | 2 | NT | NT | 6 | NT | NT | 3 | NT | NT | 0 | NT |
|  | - CC398 MRSA isolates | 0 | NT | NT | 1 | NT | NT | 2 | NT | NT | 0 | NT |
|  | - non-CC398 MRSA isolates | 1 | NT | NT | 5 | NT | NT | 1 | NT | NT | 0 | NT |
|  | - non-CC398 isolates with LA-MRSA-*spa*-types | 0 | NT | NT | 0 | NT | NT | 0 | NT | NT | 0 | NT |
|  | - non-CC398 isolates with HA/CA MRSA *spa* types, not recovered | 0 | NT | NT | 5 | NT | NT | 0 | NT | NT | 0 | NT |
|  | - non-CC398 isolates with HA/CA MRSA *spa* types, successfully re-cultured | 1 | NT | NT | 0 | NT | NT | 1 | NT | NT | 0 | NT |
|  | - MRSA isolates, not typed | 1 | NT | NT | 0 | NT | NT | 0 | NT | NT | 0 | NT |
| Broilers | Samples | 173 | NT | NT | 79 | NT | NT | 80 | NT | NT | 60 | NT |
|  | MRSA isolates | 7 | NT | NT | 2 | NT | NT | 2 | NT | NT | 2 | NT |
|  | - CC398 MRSA isolates | 5 | NT | NT | 2 | NT | NT | 2 | NT | NT | 2 | NT |
|  | - non-CC398 MRSA isolates | 2 | NT | NT | 0 | NT | NT | 0 | NT | NT | 0 | NT |
|  | - non-CC398 isolates with LA-MRSA-*spa*-types | 0 | NT | NT | 0 | NT | NT | 0 | NT | NT | 0 | NT |
|  | - non-CC398 isolates with HA/CA MRSA *spa* types, not recovered | 2 | NT | NT | 0 | NT | NT | 0 | NT | NT | 0 | NT |
|  | - non-CC398 isolates with HA/CA MRSA *spa* types, successfully re-cultured | 0 | NT | NT | 0 | NT | NT | 0 | NT | NT | 0 | NT |
|  | - MRSA isolates, not typed | 0 | NT | NT | 0 | NT | NT | 0 | NT | NT | 0 | NT |

This table shows the number of pooled nasal swab samples collected from healthy food-producing farm animals in the Belgian MRSA monitoring program, organized by the Federal Agency for the Safety of the Food Chain, between 2011 and 2021. It also shows the MRSA isolates recovered and their breakdown according to the selection criteria used in this study. Categories are nested as indicated by the indentation. In 2011 in poultry, isolates were historically gathered through two isolation methods which were combined for this table. Abbreviations: hospital-/community-associated (HA/CA), livestock-associated (LA), not targeted (NT). The data was collected from EFSA and ECDC (European Food Safety Authority and European Centre for Disease Prevention and Control), 2021. The European Union Summary Report on Antimicrobial Resistance in zoonotic and indicator bacteria from humans, animals and food in 2018/2019. EFSA Journal 2021;19(4):6490, 179 pp. <https://doi.org/10.2903/j.efsa.2021.6490>; EFSA (European Food Safety Authority) and ECDC (European Centre for Disease Prevention and Control), 2023. The European Union Summary Report on Antimicrobial Resistance in zoonotic and indicator bacteria from humans, animals and food in 2020/2021. EFSA Journal 2023; 21(3):7867, 232 pp. <https://doi.org/10.2903/j.efsa.2023.7867>; Annual reports published on the FASFC website for the data from 2011 and 2013 (<https://favv-afsca.be/fr/antibioresistance-resultats#cerva>).

## Table S2: Geographical clustering of the coordinates

| **Cluster** | **Mean distance from cluster center (km)** | **Isolates** |
| --- | --- | --- |
| A | 4.72 | VAR697, VAR702 |
| B | 15.17 | VAR708, VAR785, VAR786 |
| C | 29.02 | VAR699, VAR700, VAR701, VAR703, VAR704 |
| D | 26.68 | VAR305, VAR705, VAR706, VAR707 |
| E | 0 | VAR698 |

This table contains the results of the K-means clustering on the coordinates of the isolates. The first column lists the cluster identifier, the second column lists the mean distance to the cluster center. The third column contains the isolates that are part of the corresponding geographical cluster. No geographical data was available for isolates VAR304 and VAR307.

## Table S3: Custom Kraken 2 database

| **Branch** | **RefSeq Category** | **Assembly presentation** | **Assembly level** | **Database** |
| --- | --- | --- | --- | --- |
| Archaea | / | Full | Complete | RefSeq |
|  |  |  | Chromosome | RefSeq |
|  |  |  | Scaffold | RefSeq |
| Bacteria | / | Full | Complete | RefSeq |
| Fungi | / | Full | Complete | RefSeq |
|  |  |  | Chromosome | RefSeq |
|  |  |  | Scaffold | RefSeq |
| Protozoa | / | Full | Complete | RefSeq |
|  |  |  | Chromosome | RefSeq |
|  |  |  | Scaffold | RefSeq |
| Viruses | / | Full | Complete | RefSeq |

This table lists the reference genomes included in the in-house constructed Kraken 2 database. Assemblies containing the term “unclassified” at any taxonomic rank were excluded. The database additionally contains the human and several mammalian reference genomes.

## Table S4: Overview of the WGS datasets

| **Isolate** | **Collection date** | **Origin** | ***spa* type** | **BioSample accession** |
| --- | --- | --- | --- | --- |
| VAR304 | 2011-xx-xx^(1)^ | laying hen | t037 | SAMN56734142 |
| VAR305 | 2017-09-14 | laying hen | t037 | SAMN56734143 |
| VAR307 | 2018-03-13 | cattle (meat) | t223 | SAMN56734144 |
| VAR697 | 2012-05-24 | cattle (dairy) | t037 | SAMN56734145 |
| VAR698 | 2013-10-17 | fattening pig | t044 | SAMN56734146 |
| VAR699 | 2018-03-23 | cattle (meat) | t223 | SAMN56734147 |
| VAR700 | 2018-03-26 | cattle (dairy) | t223 | SAMN56734148 |
| VAR701 | 2018-04-17 | cattle (meat) | t223 | SAMN56734149 |
| VAR702 | 2018-10-09 | cattle (dairy) | t1257 | SAMN56734150 |
| VAR703 | 2018-10-11 | cattle (dairy) | t223 | SAMN56734151 |
| VAR704 | 2018-10-16 | cattle (dairy) | t223 | SAMN56734152 |
| VAR705 | 2021-01-22 | cattle (meat) | t037 | SAMN56734153 |
| VAR706 | 2021-01-26 | cattle (meat) | t037 | SAMN56734154 |
| VAR707 | 2021-01-27 | cattle (dairy) | t037 | SAMN56734155 |
| VAR708 | 2021-03-22 | cattle (veal calves) | t386 | SAMN56734156 |
| VAR785 | 2021-08-24 | cattle (dairy) | t037 | SAMN56734157 |
| VAR786 | 2021-08-24 | cattle (dairy) | t037 | SAMN56734158 |

The first column lists the isolate name. The second and third columns lists the collection date and isolate origin, respectively. The fourth column lists the *spa* types of the isolates, which were first determined using Sanger sequencing and then confirmed using WGS, which gave identical results. The fifth column lists the BioSample accession number of the dataset. Abbreviations: not available (n/a). Notes: (1) Isolate VAR304 was collected in 2011, but the exact date is unknown.

## Table S5: Read trimming statistics

| **Isolate** | **Read pairs in** | **Read pairs after trimming** | **Q30 rate before trimming** | **Q30 rate after trimming** |
| --- | --- | --- | --- | --- |
| VAR304 | 476,500 | 457,375 | 0.94 | 0.97 |
| VAR305 | 495,164 | 476,077 | 0.94 | 0.97 |
| VAR307 | 436,502 | 417,439 | 0.94 | 0.97 |
| VAR697 | 451,676 | 409,374 | 0.91 | 0.96 |
| VAR698 | 450,837 | 415,645 | 0.92 | 0.97 |
| VAR699 | 485,862 | 456,269 | 0.92 | 0.97 |
| VAR700 | 442,421 | 410,383 | 0.92 | 0.97 |
| VAR701 | 476,624 | 443,000 | 0.92 | 0.97 |
| VAR702 | 516,636 | 491,276 | 0.94 | 0.97 |
| VAR703 | 543,810 | 523,146 | 0.94 | 0.97 |
| VAR704 | 441,751 | 418,520 | 0.90 | 0.95 |
| VAR705 | 482,067 | 455,771 | 0.93 | 0.97 |
| VAR706 | 211,964 | 197,346 | 0.85 | 0.92 |
| VAR707 | 411,055 | 391,042 | 0.93 | 0.96 |
| VAR708 | 435,042 | 412,552 | 0.93 | 0.97 |
| VAR785 | 397,080 | 382,242 | 0.91 | 0.95 |
| VAR786 | 381,334 | 361,876 | 0.91 | 0.96 |

This table lists read trimming statistics. The first column lists the isolate name. The second and third columns list the number of read pairs before and after trimming, respectively. The fourth and fifth columns list the Q30 rate before and after trimming, respectively.

## Table S6: *De novo* assembly statistics

| **Isolate** | **Nb. of contigs** | **Total length (bp)** | **N50 (bp)** | **Median depth** | **Mapping rate (%)** |
| --- | --- | --- | --- | --- | --- |
| VAR304 | 44 | 2,887,478 | 200,555 | 70 | 99.30 |
| VAR305 | 35 | 2,890,524 | 260,493 | 71 | 99.51 |
| VAR307 | 50 | 2,822,876 | 127,038 | 63 | 99.46 |
| VAR697 | 31 | 2,886,235 | 301,412 | 65 | 98.95 |
| VAR698 | 24 | 2,768,733 | 322,981 | 70 | 99.17 |
| VAR699 | 34 | 2,825,526 | 154,970 | 74 | 99.08 |
| VAR700 | 34 | 2,823,461 | 229,175 | 66 | 98.98 |
| VAR701 | 49 | 2,837,461 | 122,042 | 69 | 98.92 |
| VAR702 | 51 | 2,931,810 | 134,086 | 76 | 99.19 |
| VAR703 | 52 | 2,833,484 | 108,122 | 82 | 98.93 |
| VAR704 | 61 | 2,829,750 | 92,574 | 62 | 99.12 |
| VAR705 | 45 | 2,883,207 | 174,950 | 71 | 99.20 |
| VAR706 | 71 | 2,875,854 | 84,913 | 27 | 98.88 |
| VAR707 | 103 | 2,865,773 | 56,638 | 59 | 99.05 |
| VAR708 | 54 | 2,841,365 | 106,776 | 66 | 98.92 |
| VAR785 | 43 | 2,888,183 | 216,416 | 56 | 99.42 |
| VAR786 | 41 | 2,889,626 | 256,441 | 54 | 99.47 |

The first column lists the isolate name. The second, third, and fourth column list the number of contigs, the total assembly length, and the N50, respectively. These metrics were calculated by QUAST. The fifth and sixth column lists the median read depth and mapping rate, determined by mapping the processed reads against the assembly using Bowtie2.

## Table S7: Detected SCC*mec* types and *mecA* genes

| **Isolate** | **SCC*mec* type** | ***mecA* gene GenBank accession** |
| --- | --- | --- |
| VAR304 | III(3A) | AB037671 |
| VAR305 | III(3A) | AB037671 |
| VAR307 | IV(2B) | AB373032 |
| VAR697 | III(3A) | AB037671 |
| VAR698 | IVc(2B) | AB373032 |
| VAR699 | IV(2B) | AB373032 |
| VAR700 | IVa(2B) | AB373032 |
| VAR701 | IVa(2B) | AB373032 |
| VAR702 | IVd(2B) | AB373032 |
| VAR703 | IVa(2B) | AB373032 |
| VAR704 | IV(2B) | AB373032 |
| VAR705 | III(3A) | AB037671 |
| VAR706 | III(3A) | AB037671 |
| VAR707 | III(3A) | AB037671 |
| VAR708 | IVa(2B) | AB505628 |
| VAR785 | III(3A) | AB037671 |
| VAR786 | III(3A) | AB037671 |

This table contains the SCCmecFinder output. The first column lists the isolate name. The second and third columns list the detected SCC*mec* type and GenBank accession for the detected *mecA* gene, respectively.

## Table S8: Detected AMR genes by ResFinder4

| **ST** | **Isolate** | ***aac(6')-aph(2'')*** | ***aadD*** | ***ant(6)-Ia*** | ***ant(9)-Ia*** | ***aph(3')-III*** | ***blaZ*** | ***bleO*** | ***cat(pC194)*** | ***erm(A)*** | ***erm(C)*** | ***fexA*** | ***grlA* p.S80Y** | ***gyrA* p.S84L** | ***mecA*** | ***rpoB* p.H481N** | ***rpoB* p.I527M** | ***rpoB* p.S529L** | ***tet(K)*** | ***tet(M)*** |
| --- | --- | --- | --- | --- | --- | --- | --- | --- | --- | --- | --- | --- | --- | --- | --- | --- | --- | --- | --- | --- |
| 1 | VAR708 | 0 | 0 | 1 | 0 | 1 | 1 | 0 | 0 | 0 | 1 | 1 | 0 | 0 | 1 | 0 | 0 | 0 | 1 | 0 |
| 22 | VAR307 | 0 | 0 | 0 | 0 | 0 | 0* | 0 | 0 | 0 | 0 | 0 | 0 | 0 | 1 | 0 | 0 | 0 | 0 | 0 |
| 22 | VAR699 | 0 | 0 | 0 | 0 | 0 | 1 | 0 | 0 | 0 | 0 | 0 | 0 | 0 | 1 | 0 | 0 | 0 | 0 | 0 |
| 22 | VAR700 | 0 | 0 | 0 | 0 | 0 | 1 | 0 | 0 | 0 | 0 | 0 | 0 | 0 | 1 | 0 | 0 | 0 | 0 | 0 |
| 22 | VAR701 | 0 | 0 | 0 | 0 | 0 | 1 | 0 | 0 | 0 | 1 | 0 | 0 | 0 | 1 | 0 | 0 | 0 | 0 | 0 |
| 22 | VAR703 | 0 | 0 | 0 | 0 | 0 | 1 | 0 | 0 | 0 | 1 | 0 | 0 | 0 | 1 | 0 | 0 | 0 | 0 | 0 |
| 22 | VAR704 | 0 | 0 | 0 | 0 | 0 | 0* | 0 | 0 | 0 | 1 | 0 | 0 | 0 | 1 | 0 | 0 | 0 | 0 | 0 |
| 80 | VAR698 | 0 | 0 | 1 | 0 | 1 | 0 | 0 | 0 | 0 | 0 | 0 | 0 | 0 | 1 | 0 | 0 | 0 | 0 | 0 |
| 239 | VAR304 | 0 | 1 | 1 | 1 | 1 | 1 | 1 | 1 | 1 | 0 | 0 | 0 | 0 | 1 | 1 | 0 | 1 | 1 | 1 |
| 239 | VAR305 | 0 | 1 | 1 | 1 | 1 | 1 | 1 | 1 | 1 | 0 | 0 | 0 | 0 | 1 | 1 | 0 | 1 | 1 | 1 |
| 239 | VAR697 | 0 | 1 | 1 | 1 | 1 | 1 | 1 | 1 | 1 | 0 | 0 | 0 | 0 | 1 | 1 | 0 | 1 | 1 | 1 |
| 239 | VAR705 | 0 | 1 | 1 | 1 | 1 | 1 | 1 | 1 | 1 | 0 | 0 | 0 | 0 | 1 | 1 | 0 | 1 | 1 | 1 |
| 239 | VAR706 | 0 | 1 | 1 | 1 | 1 | 1 | 1 | 1 | 1 | 0 | 0 | 0 | 0 | 1 | 1 | 0 | 1 | 1 | 1 |
| 239 | VAR707 | 0 | 1 | 1 | 1 | 1 | 1 | 1 | 1 | 1 | 0 | 0 | 0 | 0 | 1 | 1 | 0 | 1 | 1 | 1 |
| 239 | VAR785 | 0 | 1 | 1 | 1 | 1 | 1 | 1 | 1 | 1 | 0 | 0 | 0 | 0 | 1 | 1 | 0 | 1 | 1 | 1 |
| 239 | VAR786 | 0 | 1 | 1 | 1 | 1 | 1 | 1 | 1 | 1 | 0 | 0 | 0 | 0 | 1 | 1 | 0 | 1 | 1 | 1 |
| 612 | VAR702 | 1 | 0 | 0 | 0 | 0 | 0* | 0 | 0 | 0 | 1 | 0 | 1 | 1 | 1 | 1 | 1 | 0 | 0 | 1 |

This table shows the AMR-associated genes and mutations detected by ResFinder4. A '1' indicates the presence of the corresponding gene or mutation, while a '0' indicates its absence. Isolates are grouped by ST as indicated in the first column. Discrepancies in genes detected by either ResFinder4 or AMRFinder+ are indicated with an asterisk (*) (i.e., detected by one but not the other). Note that an additional read-mapping based correction was done for the AMRFinder analysis, but not for ResFinder4. The *catA* gene reported by AMRFinder+ and *cat(pC194)* reported by ResFinder4 have very similar sequences, and its detection was therefore considered matching. The following genes/mutations detected by AMRFinder+ were not detected by ResFinder4: *blaI*, *blaPC1*, *blaR1*, *pbp4* R200L, *dfrS1*, *mecI*, *mecR1*, *folP* E208K, *folP* E258EKE, *folP* F17L, *fosB*, *murA* G257D, *sat4*, *tet*(38), and *qacC*. Note that the *grlA* pS80Y mutation is listed as *parC* pS80Y by AMRFinder+.

## Table S9: Phenotypic susceptibility testing results

|  | | | **aminoglycosides** | | | **b-lactams** | | **Phenicols** | **macrolides** | **Fluoroquinolones** | **rifamycins** | **sulphonamides** | **Tetracyclines** | **diaminopyrimidines** | **streptogramins** | **lincosamides** | **fusidanines** | **oxazolidinones** | **RNA synthetase inhibitors** | **pleuromutilines** | **glycopeptides** |
| --- | --- | --- | --- | --- | --- | --- | --- | --- | --- | --- | --- | --- | --- | --- | --- | --- | --- | --- | --- | --- | --- |
| **Isolate** | **ST** | ***spa type*** | **GEN** | **KAN** | **STR** | **PEN** | **FOX** | **CHL** | **ERY** | **CIP** | **RIF** | **SMX** | **TET** | **TMP** | **SYN** | **CLI** | **FUS** | **LZD** | **MUP** | **TIA** | **VAN** |
| VAR708 | 1 | t386 | <=0,5 | >32 | >32 | >1 | >16 | =64 | >8 | <=0,25 | <=0,015 | <=64 | >16 | <=1 | =1 | >4 | <=0,25 | =2 | <=0,5 | <=0,5 | <=1 |
| VAR307 | 22 | t223 | <=1 | <=4 | <=4 | >2 | 16 | 8 | 0.5 | <=0.25 | <=0.016 | <=64 | <=0.5 | <=2 | <=0.5 | <=0.12 | <=0.5 | 4 | <=0.5 | 1 | <=1 |
| VAR699 | 22 | t223 | <=1 | <=4 | <=4 | >2 | 8 | 8 | 0.5 | <=0.25 | <=0.016 | <=64 | <=0.5 | <=2 | <=0.5 | <=0.12 | <=0.5 | 2 | <=0.5 | 1 | <=1 |
| VAR700 | 22 | t223 | <=1 | <=4 | <=4 | >2 | 8 | 8 | 0.5 | <=0.25 | <=0.016 | <=64 | <=0.5 | <=2 | <=0.5 | <=0.12 | <=0.5 | 2 | <=0.5 | 1 | <=1 |
| VAR701 | 22 | t223 | <=1 | <=4 | <=4 | >2 | 16 | 8 | >8 | <=0.25 | <=0.016 | <=64 | <=0.5 | <=2 | <=0.5 | <=0.12 | <=0.5 | 2 | <=0.5 | 1 | <=1 |
| VAR703 | 22 | t223 | <=1 | <=4 | <=4 | >2 | 8 | 8 | >8 | <=0.25 | <=0.016 | <=64 | <=0.5 | <=2 | <=0.5 | <=0.12 | <=0.5 | 2 | <=0.5 | 1 | <=1 |
| VAR704 | 22 | t223 | <=1 | <=4 | <=4 | >2 | 8 | 8 | >8 | <=0.25 | <=0.016 | <=64 | <=0.5 | <=2 | <=0.5 | <=0.12 | <=0.5 | 2 | <=0.5 | 1 | <=1 |
| VAR698 | 80 | t044 | <=1 | >64 | >32 | 0.5 | 16 | 8 | 0.5 | <=0.25 | <=0.016 | <=64 | <=0.5 | <=2 | <=0.5 | <=0.12 | <=0.5 | 2 | <=0.5 | <=0.5 | <=1 |
| VAR304 | 239 | t037 | <=1 | >64 | >32 | >2 | >16 | 64 | >8 | <=0.25 | >0.5 | 512 | >16 | <=2 | <=0.5 | <=0.12 | <=0.5 | 2 | <=0.5 | <=0.5 | <=1 |
| VAR305 | 239 | t037 | <=1 | >64 | >32 | >2 | >16 | >64 | >8 | <=0.25 | >0.5 | >512 | >16 | <=2 | 1 | <=0.12 | <=0.5 | 2 | <=0.5 | 1 | <=1 |
| VAR697 | 239 | t037 | <=1 | >64 | >32 | >2 | >16 | 64 | >8 | <=0.25 | >0.5 | 512 | >16 | <=2 | <=0.5 | <=0.12 | <=0.5 | <=1 | <=0.5 | <=0.5 | <=1 |
| VAR705 | 239 | t037 | <=0,5 | >32 | >32 | >1 | >16 | =64 | >8 | <=0,25 | >0,5 | >512 | >16 | <=1 | <=0,5 | <=0,12 | <=0,25 | =2 | <=0,5 | <=0,5 | <=1 |
| VAR706 | 239 | t037 | <=0,5 | >32 | >32 | >1 | >16 | =64 | >8 | <=0,25 | >0,5 | >512 | >16 | <=1 | <=0,5 | <=0,12 | <=0,25 | =2 | <=0,5 | <=0,5 | <=1 |
| VAR707 | 239 | t037 | <=0,5 | >32 | >32 | >1 | >16 | =64 | >8 | <=0,25 | >0,5 | >512 | >16 | <=1 | <=0,5 | <=0,12 | <=0,25 | =2 | <=0,5 | <=0,5 | <=1 |
| VAR785 | 239 | t037 | <=0,5 | >32 | >32 | >1 | >16 | =64 | >8 | <=0,25 | >0,5 | >512 | >16 | <=1 | =1 | <=0,12 | <=0,25 | =2 | <=0,5 | <=0,5 | <=1 |
| VAR786 | 239 | t037 | <=0.5 | >32 | >32 | >1 | >16 | =64 | >8 | <=0.25 | >0.5 | >512 | >16 | <=1 | <=0.5 | <=0.12 | <=0.25 | <=1 | <=0,5 | <=0.5 | <=1 |
| VAR702 | 612 | t1257 | 16 | >64 | <=4 | >2 | >16 | 8 | >8 | >8 | >0.5 | >512 | >16 | >32 | <=0.5 | <=0.12 | <=0.5 | 2 | <=0.5 | 1 | <=1 |

This table shows the results of phenotypic susceptibility testing against various antibiotics. The first, second, and third column list the isolate name, ST, and *spa* type, respectively. The remaining columns lists the observed MIC values for the corresponding antibiotics. Abbreviations: Gentamicin (GEN), kanamycin (KAN), streptomycin (STR), Penicillin (PEN), cefoxitin (FOX), chloramphenicol (CHL), erythromycin (ERY), ciprofloxacin (CIP), rifampin (RIF), sulfamethoxazole (SMX), tetracycline (TET), trimethoprim (TMP), quinupristin/dalfopristin (SYN), clindamycin (CLI), fusidate (FUS), linezolid (LZD), mupirocin (MUP), tiamulin (TIA), and vancomycin (VAN). The red color indicates phenotypic resistance, green color indicates phenotypic susceptibility.

## Table S10: Plasmids detected by MOB-suite

| **ST** | **isolate** | **AA004** | **AA069** | **AA083** | **AA410** | **AA411** | **AA848** | **AA850** | **AB173** | **AB633** | **AB642** | **AB918** | **AC333** | **AC627** | **AC670** | **AD159** | **AD858** | **AE018** | **AE387** | **AG371** | **AG777** | **novel** |
| --- | --- | --- | --- | --- | --- | --- | --- | --- | --- | --- | --- | --- | --- | --- | --- | --- | --- | --- | --- | --- | --- | --- |
| 1 | VAR708 | 0 | 0 | 0 | 0 | 1 | 0 | 0 | 0 | 0 | 0 | 1 | 0 | 1 | 0 | 0 | 0 | 1 | 0 | 1 | 0 | 0 |
| 22 | VAR307 | 0 | 0 | 0 | 1 | 0 | 0 | 0 | 0 | 0 | 0 | 0 | 0 | 0 | 0 | 0 | 1 | 0 | 1 | 0 | 0 | 0 |
| 22 | VAR699 | 0 | 0 | 0 | 1 | 0 | 0 | 0 | 0 | 0 | 0 | 0 | 0 | 0 | 0 | 0 | 1 | 0 | 1 | 0 | 0 | 0 |
| 22 | VAR700 | 0 | 0 | 0 | 1 | 0 | 0 | 0 | 0 | 0 | 0 | 0 | 0 | 0 | 0 | 0 | 1 | 0 | 1 | 0 | 0 | 0 |
| 22 | VAR701 | 1 | 0 | 0 | 1 | 0 | 0 | 0 | 0 | 1 | 0 | 0 | 0 | 1 | 0 | 0 | 1 | 0 | 1 | 0 | 0 | 1 |
| 22 | VAR703 | 0 | 0 | 0 | 1 | 0 | 0 | 0 | 0 | 1 | 0 | 0 | 0 | 1 | 0 | 0 | 1 | 0 | 1 | 0 | 0 | 0 |
| 22 | VAR704 | 1 | 0 | 0 | 1 | 0 | 0 | 0 | 0 | 0 | 0 | 0 | 0 | 1 | 0 | 0 | 1 | 0 | 1 | 0 | 0 | 0 |
| 80 | VAR698 | 0 | 0 | 0 | 0 | 0 | 0 | 0 | 0 | 0 | 0 | 0 | 0 | 0 | 0 | 1 | 1 | 1 | 0 | 0 | 0 | 0 |
| 239 | VAR304 | 0 | 0 | 1 | 0 | 0 | 0 | 0 | 0 | 0 | 1 | 0 | 1 | 0 | 1 | 1 | 0 | 1 | 1 | 0 | 0 | 0 |
| 239 | VAR305 | 0 | 0 | 1 | 0 | 0 | 0 | 0 | 0 | 0 | 1 | 0 | 1 | 0 | 0 | 1 | 0 | 1 | 1 | 0 | 0 | 0 |
| 239 | VAR697 | 0 | 0 | 1 | 0 | 0 | 0 | 0 | 0 | 0 | 1 | 0 | 1 | 0 | 1 | 1 | 0 | 1 | 1 | 0 | 0 | 0 |
| 239 | VAR705 | 0 | 0 | 1 | 0 | 0 | 0 | 0 | 0 | 0 | 1 | 0 | 1 | 0 | 1 | 1 | 0 | 1 | 1 | 0 | 0 | 0 |
| 239 | VAR706 | 0 | 0 | 1 | 0 | 0 | 0 | 0 | 1 | 0 | 1 | 0 | 1 | 0 | 1 | 0 | 0 | 1 | 1 | 0 | 0 | 0 |
| 239 | VAR707 | 0 | 0 | 1 | 0 | 0 | 0 | 0 | 0 | 0 | 1 | 0 | 1 | 0 | 1 | 1 | 0 | 1 | 1 | 0 | 1 | 0 |
| 239 | VAR785 | 0 | 0 | 1 | 0 | 0 | 1 | 0 | 0 | 0 | 1 | 0 | 1 | 0 | 1 | 1 | 0 | 1 | 1 | 0 | 0 | 0 |
| 239 | VAR786 | 0 | 1 | 1 | 0 | 0 | 0 | 0 | 0 | 0 | 1 | 0 | 1 | 0 | 1 | 1 | 0 | 1 | 1 | 0 | 0 | 0 |
| 612 | VAR702 | 0 | 0 | 0 | 0 | 0 | 0 | 1 | 0 | 0 | 0 | 0 | 0 | 1 | 0 | 0 | 0 | 1 | 1 | 0 | 0 | 0 |

This table lists the results of screening the plasmids with MOB-suite. The first and second columns list the sequence type and the isolate name, respectively. The remaining columns indicate if the plasmid with the given primary identifier was present in the corresponding dataset. These identifiers correspond to the ‘primary id’ field in the MOB-suite plasmid database, except for the last column which corresponds to a plasmid that was not yet present in the database. A ‘1’ indicates that the corresponding plasmid was present, and ‘0’ indicates that it was absent.

## Table S11: Number of assemblies from AllTheBacteria with matching STs

| **ST** | **# In-house** | **# AllTheBacteria** |
| --- | --- | --- |
| 1 | 1 | 4,765 |
| 22 | 6 | 12,101 |
| 80 | 1 | 566 |
| 239 | 8 | 3094 |
| 612 | 1 | 41 |

This table lists the number of datasets per ST. The first column lists the sequence type. The second and third columns lists the number of genomes for the in-house and AllTheBacteria datasets respectively.

## Table S12: Detected AMR genes and mutations by NCBI AMRFinder

| **ST** | **Isolate** | **AMG** | | | | | ***BLA*** | | | | | | | | ***BLE*** | ***CHL*** | | ***FOF*** | | ***ML*** | | ***QA*** | ***QN*** | | ***RIF*** | | | ***ST*** | ***SUL*** | | | ***TET*** | | | ***TMP*** | |
| --- | --- | --- | --- | --- | --- | --- | --- | --- | --- | --- | --- | --- | --- | --- | --- | --- | --- | --- | --- | --- | --- | --- | --- | --- | --- | --- | --- | --- | --- | --- | --- | --- | --- | --- | --- | --- |
|  |  | ***aac(6')-Ie / aph(2'')-Ia*** | ***aadD1*** | ***ant(6)-Ia*** | ***ant(9)-Ia*** | ***aph(3')-IIIa*** | ***blaI*** | ***blaPC1*** | ***blaR1*** | ***blaZ*** | ***mecA*** | ***mecI*** | ***mecR1*** | ***pbp4 R200L*** | ***bleO*** | ***catA*** | ***fexA*** | ***fosB*** | ***murA G257D*** | ***erm(A)*** | ***erm(C)*** | ***qacC*** | ***gyrA S84L*** | ***parC S80Y*** | ***rpoB H481N*** | ***rpoB I527M*** | ***rpoB S529L*** | ***sat4*** | ***folP E208K*** | ***folP E258EKE*** | ***folP F17L*** | ***tet(38)*** | ***tet(K)*** | ***tet(M)*** | ***dfrS1*** | ***dfrB A135T*** |
| ST1 | VAR708 | 0 | 0 | P | 0 | P | P | 1^(1)^ | P | P | 1 | 0 | 0 | 1 | 0 | 0 | 1 | 0 | 0 | 0 | P | 0 | 0 | 0 | 0 | 0 | 0 | P | 0 | 0 | 0 | 1 | 1 | 0 | 0 | 0 |
| ST22 | VAR307 | 0 | 0 | 0 | 0 | 0 | 1^(1)^ | 1^(1)^ | 0 | 1^(1)^ | 1 | 0 | 0 | 0 | 0 | 0 | 0 | 0 | 0 | 0 | 0 | 0 | 0 | 0 | 0 | 0 | 0 | 0 | 0 | 0 | 0 | 1 | 0 | 0 | 0 | 1 |
| ST22 | VAR699 | 0 | 0 | 0 | 0 | 0 | 1 | 1^(1)^ | 0 | 1 | 1 | 0 | 0 | 0 | 0 | 0 | 0 | 0 | 0 | 0 | 0 | 0 | 0 | 0 | 0 | 0 | 0 | 0 | 0 | 0 | 0 | 1 | 0 | 0 | 0 | 1 |
| ST22 | VAR700 | 0 | 0 | 0 | 0 | 0 | 1 | 1^(1)^ | 0 | 1 | 1 | 0 | 0 | 0 | 0 | 0 | 0 | 0 | 0 | 0 | 0 | 0 | 0 | 0 | 0 | 0 | 0 | 0 | 0 | 0 | 0 | 1 | 0 | 0 | 0 | 1 |
| ST22 | VAR701 | 0 | 0 | 0 | 0 | 0 | 1 | 1^(1)^ | 0 | 1 | 1 | 0 | 0 | 0 | 0 | 0 | 0 | 0 | 0 | 0 | P | P | 0 | 0 | 0 | 0 | 0 | 0 | 0 | 0 | 0 | 1 | 0 | 0 | 0 | 1 |
| ST22 | VAR703 | 0 | 0 | 0 | 0 | 0 | 1 | 1^(1)^ | 0 | 1 | 1 | 0 | 0 | 0 | 0 | 0 | 0 | 0 | 0 | 0 | P | P | 0 | 0 | 0 | 0 | 0 | 0 | 0 | 0 | 0 | 1 | 0 | 0 | 0 | 1 |
| ST22 | VAR704 | 0 | 0 | 0 | 0 | 0 | 1 | 1^(1)^ | 0 | 1^(1)^ | 1 | 0 | 0 | 0 | 0 | 0 | 0 | 0 | 0 | 0 | P | 0 | 0 | 0 | 0 | 0 | 0 | 0 | 0 | 0 | 0 | 1 | 0 | 0 | 0 | 1 |
| ST80 | VAR698 | 0 | 0 | P | 0 | P | 0 | 0 | 0 | 0 | 1 | 0 | 0 | 0 | 0 | 0 | 0 | 0 | 0 | 0 | 0 | 0 | 0 | 0 | 0 | 0 | 0 | P | 0 | 0 | 0 | 1 | 0 | 0 | 0 | 0 |
| ST239 | VAR304 | 0 | P | P | P | P | 1 | 1 | 1 | P | 1 | 0^(2)^ | 1 | 0 | P | P | 0 | 1 | 1 | P | 0 | 0 | 0 | 0 | 1 | 0 | 1 | P | 0 | 1 | 1 | 1 | P | 1 | 0 | 0 |
| ST239 | VAR305 | 0 | P | P | P | P | 1 | 1^(1)^ | 1 | P | 1 | 0^(2)^ | 1 | 0 | P | P | 0 | 1 | 1 | P | 0 | 0 | 0 | 0 | 1 | 0 | 1 | P | 0 | 1 | 1 | 1 | P | 1 | 0 | 0 |
| ST239 | VAR697 | 0 | P | P | 1 | P | 1 | 1^(1)^ | 1 | P | 1 | 0^(2)^ | 1 | 0 | P | P | 0 | 1 | 1 | 1 | 0 | 0 | 0 | 0 | 1 | 0 | 1 | P | 0 | 1 | 1 | 1 | P | 1 | 0 | 0 |
| ST239 | VAR705 | 0 | P | P | P | P | P | 1^(1)^ | P | P | 1 | 0^(2)^ | 1 | 0 | P | P | 0 | 1 | 1 | P | 0 | 0 | 0 | 0 | 1 | 0 | 1 | P | 0 | 1 | 1 | 1 | P | 1 | 0 | 0 |
| ST239 | VAR706 | 0 | P | P | 1 | P | P | 1^(1)^ | P | P | P | 0^(2)^ | P | 0 | P | P | 0 | 1 | 1 | 1 | 0 | 0 | 0 | 0 | 1 | 0 | 1 | P | 0 | 1 | 1 | 1 | P | 1 | 0 | 0 |
| ST239 | VAR707 | 0 | P | P | P | P | 1^(1)^ | 1^(1)^ | 1^(1)^ | 1^(1)^ | 1 | 0^(3)^ | 1 | 0 | P | P | 0 | 1 | 1 | P | 0 | 0 | 0 | 0 | 1 | 0 | 1 | P | 0 | 1 | 1 | 1 | P | 1 | 0 | 0 |
| ST239 | VAR785 | 0 | P | P | P | P | P | 1^(1)^ | 1^(1)^ | 1^(1)^ | 1 | 0^(2)^ | 1 | 0 | P | P | 0 | 1 | 1 | P | 0 | 0 | 0 | 0 | 1 | 0 | 1 | P | 0 | 1 | 1 | 1 | P | 1 | 0 | 0 |
| ST239 | VAR786 | 0 | P | P | P | P | P | 1 | P | P | 1 | 0^(2)^ | 1 | 0 | P | P | 0 | 1 | 1 | P | 0 | 0 | 0 | 0 | 1 | 0 | 1 | P | 0 | 1 | 1 | 1 | P | 1 | 0 | 0 |
| ST612 | VAR702 | P | 0 | 0 | 0 | 0 | 1 | 1^(1)^ | 1^(1)^ | 1^(1)^ | 1 | 0 | 0 | 0 | 0 | 0 | 0 | 1 | 1 | 0 | P | 0 | 1 | 1 | 1 | 1 | 0 | 0 | 1 | 0 | 1 | 1 | 0 | 1 | P | 0 |

*Notes: (1): Genes were not detected by the alignment based-approach but manual investigation of mapped reads revealed their presence (See Supplementary Figures S1-S4). (2): A premature stop codon was detected in the mecI gene at amino-acid position 68, which results in increased expression of mecA. (3) The mecI gene was not detected in VAR707 due to insufficient coverage. Some reads mapped to the region but the presence or absence of a premature stop codon could not be confirmed.*

# Figures

## Figure S1: Selection of the number of geographical clusters (k)

This figure shows the average silhouette width (left) and the total within-cluster sum of squares (right) for Ward clustering of isolate coordinates at different values of k. The silhouette width is maximized at k = 5, as indicated by the blue dashed line. This k-value also coincides with the elbow of the within-cluster sum of squares and was therefore selected as the number of geographical clusters.

## Figure S2: Visualization of the alignment of the processed reads against the *blaI* gene

## *blaI*

## ST22

## *blaI*

## ST239

## *blaI*

## ST1, ST80, and ST612

Isolate names are shown on the left. Coverage is indicate in the track labelled as ‘Coverage’, and the reads are shown below the coverage (and junctions) tracks. Mismatches to the database sequences are colored. Reads were mapped to the reference sequences with Bowtie2 2.4.1 with the ‘--very-sensitive-option’ enabled. The resulting BAM files were visualized using IGV 2.13.2.

## Figure S3: Visualization of the alignment of the processed reads against the *blaPC1* gene

## *blaPC1*

## ST22

## *blaPC1*

## ST239

## *blaPC1*

## ST1, ST80, and ST612

Isolate names are shown on the left. Coverage is indicate in the track labelled as ‘Coverage’, and the reads are shown below the coverage (and junctions) tracks. Mismatches to the database sequences are colored. Reads were mapped to the reference sequences with Bowtie2 2.4.1 with the ‘--very-sensitive-option’ enabled. The resulting BAM files were visualized using IGV 2.13.2. Note that isolates for which the gene was detected using the alignment-based approach were also included in the visualization.

## Figure S4: Visualization of the alignment of the processed reads against the *blaR1* gene

## *blaR1*

## ST22

## *blaR1*

## ST239

## *blaR1*

## ST1, ST80, and ST612

Isolate names are shown on the left. Coverage is indicate in the track labelled as ‘Coverage’, and the reads are shown below the coverage (and junctions) tracks. Mismatches to the database sequences are colored. Reads were mapped to the reference sequences with Bowtie2 2.4.1 with the ‘--very-sensitive-option’ enabled. The resulting BAM files were visualized using IGV 2.13.2. Note that while the gene was covered in the ST22 datasets, there were too many mismatches for it to be considered a match.

## Figure S5: Visualization of the alignment of the processed reads against the *blaZ* gene

## *blaZ*

## ST22

## *blaZ*

## ST239

## *blaZ*

## ST1, ST80, and ST612

Isolate names are shown on the left. Coverage is indicate in the track labelled as ‘Coverage’, and the reads are shown below the coverage (and junctions) tracks. Mismatches to the database sequences are colored. Reads were mapped to the reference sequences with Bowtie2 2.4.1 with the ‘--very-sensitive-option’ enabled. The resulting BAM files were visualized using IGV 2.13.2.

## Figure S6: Core-genome MLST phylogeny for ST1 (in-house + subset of public data)

Core-genome phylogeny for a subset of the isolates assigned to ST1. Note that this visualization includes only 18 of the 4,764 ST1 isolates in the AllTheBacteria database. These isolates were selected based on their relatively short allele distances to the in-house isolate. The color corresponds to the host species (see legend). The in-house isolate is highlighted in red.

## Figure S7: Core-genome MLST phylogeny for ST1 (in-house + all public data)

Core-genome phylogeny for the isolates assigned to ST1. The color corresponds to the host species (see legend). The in-house isolate is highlighted in red. The red boxes indicate clades consisting exclusively of isolates collected from *Bos taurus*, or for which host information is missing. The black arrow points to the VAR708 isolate collected in this study, which did not cluster within one of these two aforementioned clades. A subtree containing isolates similar to VAR708 is shown in Figure S5.

## Figure S8: Core-genome MLST phylogeny for ST22 (in-house + subset public data)

Core-genome phylogeny for a subset of the isolates assigned to ST22. Note that this visualization includes only 15 of the 12,095 ST22 isolates in the AllTheBacteria database. These isolates were selected based on their relatively short allele distances to the in-house isolates. The color corresponds to the host species (see legend). The in-house isolates are located at the bottom left.

## Figure S9: Core-genome MLST phylogeny for ST80 (in-house + subset public data)

Core-genome phylogeny for a subset of the isolates assigned to ST80. The color corresponds to the host species (see legend). The in-house isolate is highlighted in red.
